# Effectiveness and safety of xingnaojing injection within 24 hours for acute ischemic stroke: a multicenter prospective cohort study (TRACE)

**DOI:** 10.3389/fphar.2026.1697607

**Published:** 2026-08-12

**Authors:** Yuanyuan Chen, Dahe Qi, Siyao Che, Zangwei Chang, Dixiang Zhong, Yunlong Hao, Ye Guo, Lingbo Kong

**Affiliations:** Dongzhimen Hospital, Beijing University of Chinese Medicine, Beijing, China

**Keywords:** acute ischemic stroke (AIS), early neurological deterioration (END), prospective cohort study, traditional Chinese medicine, xingnaojing injection (XNJI)

## Abstract

**Background:**

Acute ischemic stroke (AIS) remains a leading cause of death and disability in China. Most AIS patients are ineligible for reperfusion therapies owing to the narrow therapeutic time window and risk of bleeding complications. Xingnaojing injection (XNJI) is the only traditional Chinese medicine injection that is formally approved for prehospital stroke care. Nevertheless, high-quality real-world evidence regarding its effectiveness and safety for early use within 24 h after stroke onset remains scarce. Therefore, this prospective study aimed to explore the correlations between early XNJI administration within 24 h of symptom onset and clinical outcomes in AIS patients.

**Methods:**

This is a multicenter prospective observational cohort study. In total, 512 AIS patients were enrolled from 10 tertiary hospitals in Beijing and were grouped according to whether XNJI was initiated within 24 h of symptom onset, split into an exposed group (XNJI combined with standard care, n = 208) and a non-exposed group (standard care alone, n = 304). The primary endpoint was early neurological deterioration (END), defined as a ≥2-point elevation in NIHSS score within 72 h post-onset. Inverse probability weighting (IPW) was used to balance baseline covariates. Following IPW adjustment, we further adjusted models for time-updated in-hospital and post-discharge concomitant treatment covariates to partially attenuate dynamic confounding during follow-up.

**Results:**

In the IPW-adjusted models, early XNJI administration within 24 h after the onset of AIS was associated with a reduced risk of END (adjusted odds ratio [aOR] = 0.46, 95% CI: 0.25–0.88; P = 0.018; E-value = 3.73). After adjustment, longitudinal analyses revealed significant time-by-group interactions for neurological function, functional independence and independent ADL (all P < 0.05), while the overall between-group main effects were non-significant (all P > 0.05). The two groups exhibited divergent recovery trajectories: patients receiving XNJI showed a correlative trend of more rapid improvement in neurological function at Days 3 and 10 (adjusted β ranged from −0.97 to −0.82; all P < 0.05). The XNJI group was also correlated with higher odds of achieving functional independence (modified Rankin Scale [mRS]≤2) at 1, 2, and 3 months (aOR range: 2.18–2.25; all P < 0.05) and independent activities of daily living (ADL) (Barthel Index [BI]≥90) at 2 and 3 months (aOR range: 1.49–1.62; all P < 0.05). Additionally, XNJI administration was also correlated with a reduced risk of abnormalities in neutrophil percentage (NE%) and D-dimer (all P < 0.05). No significant correlations were observed between XNJI use and in-hospital mortality, systolic blood-pressure (SBP) variation, or the risk of hepatorenal dysfunction (all P > 0.05).

**Conclusion:**

This real-world observational cohort study showed that early XNJI administration within 24 h of AIS onset reduced the risk of END. Patients receiving early XNJI intervention achieved faster recovery in acute neurological function and long-term functional outcomes; however, no sustained overall therapeutic benefit was observed across all follow-up periods compared with standard stroke care alone. No significant associations were observed between XNJI and elevated risks of in-hospital mortality, SBP fluctuation or hepatorenal dysfunction. The present study only reveals observational associations, and its definite clinical efficacy and safety remain to be further verified in subsequent Randomized Controlled Trials (RCTs).

**Trial Registration:**

ClinicalTrials.gov, identifier NCT04275349.

## 1 Introduction

Stroke remains the leading cause of adult mortality and long-term disability in China, and acute ischemic stroke (AIS) accounts for over 70% of all stroke cases (GBD, 2021 Stroke Risk Factor Collaborators, 2024; Stroke Prevention Project and Ji, 2025). In cases of AIS, early neurological deterioration (END) represents a severe acute complication, defined as an increase of ≥2 points in the National Institutes of Health Stroke Scale (NIHSS) score within 72 h of symptom onset, with an incidence of 5%–40% (Birschel et al., 2004). END is independently associated with an approximately 45% increase in 3-month disability and mortality rates among AIS patients (Thanvi et al., 2008; Geng et al., 2017). As standard reperfusion regimens for AIS, intravenous thrombolysis and endovascular treatment are constrained by a narrow therapeutic window (<4.5 h), strict imaging-based eligibility criteria, and the risk of symptomatic intracranial hemorrhage (sICH) (Tang et al., 2023; Liu et al., 2025). These two therapies currently show extremely low clinical utilization rates of only 2.76% and 3.23% in China, respectively (Modrego, 2019; Soni et al., 2021), leaving more than 90% of AIS patients ineligible for standard reperfusion treatment and highlighting an urgent clinical demand for safe and effective early adjuvant interventions for AIS.

Xingnaojing Injection (XNJI) is a sterile aqueous traditional Chinese medicine (TCM) injectable formulation refined from Angong Niuhuang Wan, a classic emergency TCM prescription with over 200 years of clinical application. Approved by the National Medical Products Administration, XNJI is the only officially authorized TCM injection indicated for prehospital emergency management of both ischemic and hemorrhagic stroke (Deng et al., 2010), and its clinical administration for acute stroke emergencies has been recommended across multiple authoritative clinical guidelines and expert consensus documents (Fang et al., 2018; Liu et al., 2010; Chinese Research Hospital Association and Chinese Association of Integrative Medicine, 2019). Preclinical pharmacological experiments have confirmed that XNJI exerts multi-target neuroprotective properties in animal models, including alleviating cerebral edema, inhibiting neuroinflammation and neuronal apoptosis, improving cerebral microcirculation, and generating mild antiplatelet effects (Qi et al., 2020; Zhang W. et al., 2021; Song et al., 2018). Accumulated clinical trials suggest correlative trends that XNJI alleviates neurological impairment, boosts perfusion within the ischemic penumbra, and rectifies dysregulated inflammatory and coagulation-related biomarkers (Jia et al., 2020; Zhang B. H. et al., 2021). Moreover, its neuroprotective and microcirculation-improving effects can be further enhanced when combined with intravenous thrombolysis (Hu et al., 2026; Cheng et al., 2021; Dong et al., 2021). Nevertheless, evidence assessments performed per the AMSTAR-2 and GRADE rating systems reveal that existing relevant clinical research is constrained by limited sample sizes, inadequate blinding protocols and substantial between-study heterogeneity, leading to generally low-grade evidence quality (Tian et al., 2021; Wang et al., 2021). Critically, large-scale real-world cohort research specifically exploring XNJI initiation within 24 h after AIS onset is still lacking.

The clinical use of XNJI is strictly regulated under national pharmaceutical laws and quality specifications. Per the revised drug instructions (China Food and Drug Administration, 2015), relevant expert consensus (Chinese Research Hospital Association and Chinese Association of Integrative Medicine, 2019), and published pharmacovigilance data (Zhang et al., 2020), adverse reactions of XNJI may involve multiple organ systems, with an overall incidence of approximately 0.26%, suggesting a favorable safety profile that still requires close clinical monitoring. Mild adverse manifestations mainly include rash, pruritus, nausea and dizziness, whereas severe adverse events consist of chest tightness, dyspnea, palpitations, chills and even anaphylactic shock. Roughly 70% of adverse events develop within the first 30 min of infusion, predominantly attributable to inappropriate medication practices such as pre-existing allergic diathesis, off-label administration, overdose, excessive infusion velocity and prolonged treatment duration (Zou et al., 2014; Tang and Ning, 2015). Regarding drug-drug interactions, robust human pharmacokinetic data for XNJI combined with alteplase, statins or antiplatelet agents are insufficient (Duan, 2021; Liang, 2020). However, multiple large-scale post-marketing safety reassessments (Chen and Dong, 2015) and meta-analyses (Zha et al., 2023; Hu et al., 2026; He et al., 2017) confirm that standardized XNJI application with intensive monitoring during the initial 30 min of infusion, as an adjuvant combined with conventional stroke therapies, does not elevate the risk of adverse outcomes including sICH, featuring an overall manageable benefit-risk profile (Guo et al., 2022).

Despite these advances, two critical evidence gaps remain regarding early XNJI administration within 24 h of AIS onset. First, high-quality clinical evidence is still limited: although systematic reviews have identified the first 72 h (particularly the initial 6 h) as the optimal therapeutic window (Tian et al., 2021; Wang et al., 2022), well-designed studies specifically exploring the efficacy of XNJI in END prevention and long-term functional improvement within the 24-h early time window remain insufficient. Second, real-world safety data (including blood pressure stability and hepatorenal function) for early XNJI use remain insufficient. This multicenter prospective observational cohort study was conducted to address these gaps. We prospectively evaluated the associations between early XNJI initiation (within 24 h of AIS onset) and clinical outcomes (with END as the primary endpoint) under routine clinical settings. Our findings aim to provide preliminary real-world references for clinical decision-making of XNJI use in pre-hospital and emergency stroke care.

## 2 Methods

### 2.1 Medicinal materials and formulation

XNJI comprises four taxonomically authenticated crude drug materials: *Moschus berezovskii Flerov* (Cervidae; animal-derived musk gland secretion), *Dryobalanops aromatica Gaertn. f.* (Dipterocarpaceae; plant resin conventionally utilized as a natural borneol source), *Curcuma aromatica Salisb.* (Zingiberaceae; dried plant rhizome), and *Gardenia jasminoides J. Ellis* (Rubiaceae; ripe dried fruit). Its water-soluble and volatile constituents are isolated by steam distillation to prepare formulations suitable for intravenous infusion (Deng et al., 2010). XNJI is manufactured by the GMP-certified pharmaceutical enterprise Wuxi Jiyu Shanhe Pharmaceutical Co., Ltd. in Wuxi, China, which strictly complies with China’s National Drug Standard (WS3-B-3353-98-2003) to guarantee stable and consistent batch-to-batch quality. The standardized manufacturing procedure is described below. First, 30 g of Curcumae Radix and 30 g of Gardeniae Fructus are steam-distilled in 1,500 mL purified water to yield 1,000 mL distillate; 7.5 g of Moschus together with 250 mL distilled water is supplemented, followed by collection of an additional 1,000 mL distillate. Next, 1 g synthetic borneol (a blend of borneol, isoborneol and camphor) and 8 g polysorbate 80 are triturated and blended into the pooled distillate. Lastly, 8 g sodium chloride is dissolved into the liquid; the resultant solution is agitated, refrigerated overnight, filtered, filled into vials and sterilized. Synthetic borneol acts as the core semi-volatile quality control marker, with a mandatory lower limit of 0.7 g/L specified under NMPA specifications (Pharmacopoeia Commission of the Ministry of Health of the People’s Republic of China, 1998; China Food and Drug Administration, 2003). In line with the ConPhyMP-recommended best-practice guidelines for complex injectable formulations (Heinrich et al., 2022), XNJI’s chemical batch consistency is validated via three orthogonal analytical approaches. GC-FID equipped with a DB-FFAP column quantifies four predominant semi-volatile markers per an established analytical protocol (Fang et al., 2012). HPLC-UV analysis using a Thermo BDS Hypersil C_18_ column (mobile phase: acetonitrile–0.02% aqueous formic acid; detection wavelength: 254 nm) generates a characteristic fingerprint consisting of 11 diagnostic peaks; curdione and germacrone are designated as index markers for Curcumae Radix, and the similarity index across all production batches remains ≥0.95 (Yang et al., 2016). UHPLC-HRMS coupled to Q-TOF mass spectrometry (Liu, 2022) further characterizes signature constituents such as curcumol, germacrone and muscone to supplement the above analytical datasets. Collectively, these corroborated analytical techniques map the bulk detectable chemical profile of XNJI and satisfy ConPhyMP-based multi-attribute characterization criteria; full compliance documentation is available in Supplementary Material S1.

### 2.2 Study design and population

This was a multicenter prospective observational cohort study based on a standardized stroke registry database. We predefined core study components, including eligibility criteria, intervention grouping, covariate adjustment strategy, 3-month follow-up period, and primary and secondary endpoints, to reduce selection bias and improve analytical reliability. To balance baseline characteristics between groups, inverse probability weighting (IPW) was applied. To mitigate time-dependent confounding, we collected all observable time-updated concomitant treatment covariates across the whole study period and adjusted for them in statistical models. These covariates covered in-hospital antithrombotic, antihypertensive, lipid-lowering and glucose-lowering therapies, concomitant traditional Chinese medicines, as well as post-discharge maintenance medications. Nevertheless, conventional IPW combined with covariate adjustment cannot fully eliminate complex time-dependent confounding derived from unmeasured dynamic factors, such as real-time fluctuations in disease severity and individualized treatment adjustments based on daily patient conditions, which remains an inherent limitation of this observational study.

AIS patients transported via 120 emergency services or presenting spontaneously to 10 Grade A tertiary hospitals in Beijing between September 2020 and June 2024 were screened for eligibility. Participants were enrolled if symptom onset occurred within 24 h, AIS diagnosis was verified by computed tomography (CT) or magnetic resonance imaging (MRI), age was ≥18 years, and written informed consent was obtained from patients or their legal representatives. Patients were excluded if they suffered from diseases causing irreversible motor dysfunction (e.g., intermittent claudication, severe osteoarthritis, rheumatoid arthritis, or gouty arthritis), concomitant organic diseases or psychotic disorders hindering scheduled outcome evaluation and follow-up, confirmed pregnancy or lactation, or non-acute cerebrovascular lesions identified by CT/MRI inconsistent with acute ischemic stroke.

### 2.3 Grouping and intervention

Patients were divided into two groups according to real-world clinical practice: the exposed group (n = 208) received XNJI treatment initiated within 24 h after symptom onset, whereas the non-exposed group (n = 304) did not receive XNJI throughout hospitalization. The routine XNJI regimen consisted of 20 mL XNJI diluted in 250 mL 0.9% saline for intravenous infusion, administered once or twice daily. The dosage and treatment duration were individually determined by attending physicians based on stroke severity and patient tolerance, with no unified fixed protocol applied in this real-world cohort. All participants received guideline-recommended standard stroke treatment (Chinese Society of Neurology and Chinese Stroke Society, 2018). To avoid time-dependent crossover bias, patients who initiated XNJI beyond 24 h after onset or switched treatment groups during hospitalization were excluded from the final analysis. All dynamically adjusted concomitant medications during follow-up were incorporated as time-varying covariates to further control confounding.

### 2.4 Sample-size calculation

Sample size calculation was performed using END as the primary outcome, defined as a ≥2-point increase in the total NIHSS score within 72 h after stroke onset. Pooled END incidence among patients receiving standard AIS care ranged from 11.9% to 18.7% according to two previous cohort studies (Zhou and Liu, 2026; Nam et al., 2021). To avoid underestimation of the required sample size, we adopted a conservative hypothesis: the expected END incidence was 10% in the exposed group and 20% in the non-exposed group. Assuming a 1:1 allocation ratio, the minimum total sample size required was 459 patients. Considering an expected 10% loss to follow-up, a common issue in prospective cohort studies, as well as potential confounding effects in real-world clinical settings, the target sample size was increased to 505 patients.

### 2.5 Data collection

Follow-up lasted for 90 days from stroke onset; detailed assessment timings and collected variables are summarized in Table 1.

**TABLE 1:** Schedule of data collection and corresponding assessment items.

Data collection approach	Study time point	Collected assessments
On-site visit	T1 (Prehospital phase, if applicable)	Demographics (age, sex, BMI), stroke onset time, prehospital medications (including XNJI), and vital signs; collected by emergency ambulance staff when available
	T2 (Baseline, within 24 h of onset)	Demographics, onset time, comorbidities, SBP, laboratory tests (WBC, NE%, D-dimer, Cr, ALT), brain imaging, plus baseline NIHSS and mRS measurement
	T3 (Day 3 post-onset)	Vital signs (including SBP) and NIHSS assessment
	T4 (Day 10 post-onset or day of discharge)	Vital signs (including SBP), laboratory markers (NE%, D-dimer, ALT, Cr), imaging data, in-hospital treatment regimens, in-hospital mortality, and NIHSS evaluation
Follow-up (telephone or in-person visit)	T5 (Day 30 ± 3 days post-onset)	mRS and BI assessment
	T6 (Day 60 ± 3 days post-onset)	mRS and BI assessment
	T7 (Day 90 ± 7 days post-onset)	mRS, BI, and post-discharge medication information

### 2.6 Outcome measures

Definitions of the outcome measures for this prospective cohort are shown in Table 2.

**TABLE 2:** Definitions of efficacy and safety outcome measures.

Outcome category	Definition	Study time points
Primary efficacy outcome	END incidence (≥2-point increment in NIHSS score)	T1 to T2 (within 3 days after symptom onset)
Secondary efficacy outcomes	Neurological function (NIHSS score)	T1 (baseline), T2 (Day 3), T3 (Day 10/discharge)
	Rates of functional independence (mRS ≤ 2)	T4 (1 month), T5 (2 months), T6 (3 months)
	Rates of independent ADL (BI ≥ 90)	T4 (1 month), T5 (2 months), T6 (3 months)
	Abnormality rates of inflammatory and coagulation indicators (NE%, D-dimer)	T1 (baseline), T3 (Day 10/discharge)
Safety outcomes	All-cause in-hospital mortality	T1 to T3 (during hospitalization)
	SBP trajectory	T1 (baseline), T2 (Day 3), T3 (Day 10/discharge)
	Hepatic (ALT) and renal (Cr) dysfunction	T1 (baseline), T3 (Day 10/discharge)

### 2.7 Data management and quality control

Prehospital data were entered in real time by ambulance physicians via electronic case report forms. In-hospital data were collected via face-to-face scale administration and abstracted from inpatient medical charts by trained researchers. All data were administered through an electronic data capture (EDC) system equipped with a dual verification framework: (1) remote real-time inspection via embedded logical validation rules to flag anomalous data entries; (2) on-site source-data audits conducted by clinical research associates (CRAs) to confirm the authenticity, accuracy and timeliness against original medical records.

### 2.8 Study population and missing data handling

In total, 621 suspected stroke patients were screened. After excluding 39 ineligible cases and 70 patients with missing critical baseline data (i.e., missing at least 2 of 3 key indicators: NIHSS score, demographic information, and medical history), 512 patients (208 in the exposed group and 304 in the non-exposed group) were included for baseline and in-hospital mortality analyses.

Follow-up status was as follows:

- By day 3 post-onset, 14 cases were lost to follow-up (LTFU) with 0 deaths, resulting in 498 patients evaluable for END.
- By day 10 or discharge, 39 cases were lost to follow-up with 8 deaths, leaving 465 patients evaluable for assessments of NIHSS, inflammatory markers, systolic blood pressure (SBP), and hepatic/renal function.
- By 1 month, 20 previously lost-to-follow-up patients were recontacted, with cumulative losses to follow-up reaching 53 cases and 9 deaths, for a total of 450 evaluable patients.
- By 2 months, 3 additional previously lost-to-follow-up patients were recontacted, with a total of 54 cases lost to follow-up and 12 deaths, leaving 446 evaluable patients.
- By 3 months, 32 more previously lost-to-follow-up patients were recontacted, bringing cumulative losses to follow-up to 26 cases and total deaths to 18 (including 6 new deaths), finally resulting in 468 evaluable patients.

**FIGURE 1:**
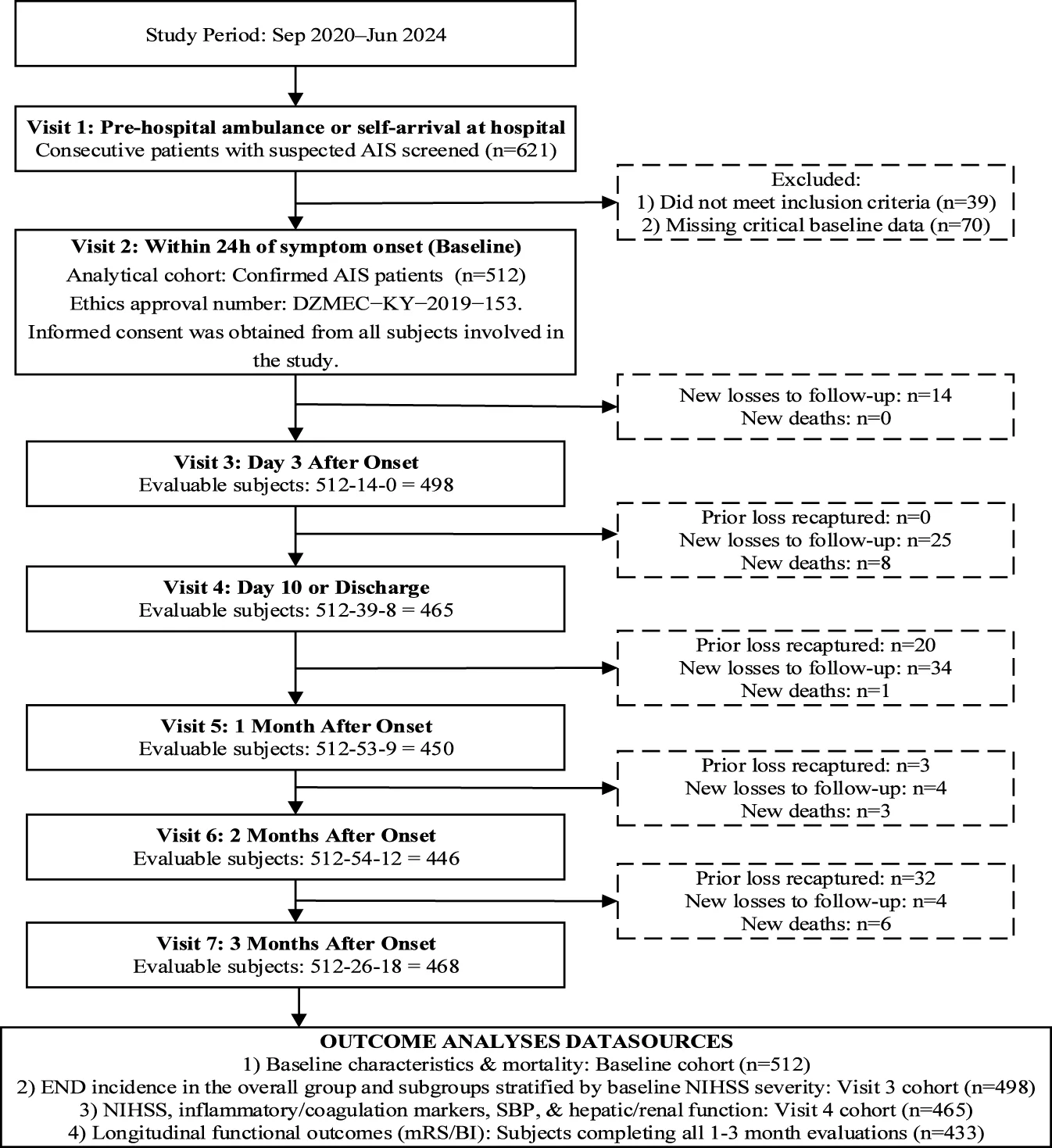
Patient Screening and Enrollment Flowchart. Flowchart depicts cohort derivation (September 2020–June 2024). Of 621 screened patients, 512 met inclusion criteria after exclusions. Serial assessments occurred at designated visits: baseline (V2; ≤24 h post-onset), day 3 (V3), day 10/discharge (V4), and 1-/2-/3-month follow-ups (V5–V7). Evaluable populations for key outcomes are annotated. Ethics: DZMEC-KY-2019-153; informed consent obtained. Abbreviations: AIS, acute ischemic stroke; XNJI, Xingnaojing Injection; NIHSS, National Institutes of Health Stroke Scale; mRS, modified Rankin Scale; BI, Barthel Index.

The patient screening and enrollment flowchart is presented in Figure 1.

Per intention-to-treat principles, deceased patients were assigned modified Rankin Scale (mRS) = 6 and Barthel Index [BI] = 0 at all subsequent time points. NIHSS analysis included only survivors, whereas longitudinal mRS/BI analysis required complete 1-/2-/3-month data (n = 433). Among all 57 collected variables, baseline BMI had the highest missing rate (14.65%). Missing pattern analysis showed that missing values were dominated by univariate isolated missingness, with a small proportion of joint missing cases, and the dataset did not present strict monotonic missingness caused by simple follow-up dropout. The key outcome indicators including NIHSS, mRS and BI maintained a data completeness rate higher than 95%. We used multiple imputation by chained equations (MICE) to handle missing covariates. The imputation model included a comprehensive set of predictors: baseline demographics, comorbidities, laboratory indices, longitudinal outcome variables, and all treatment factors during hospitalization and after discharge. Missing values were estimated according to inter-variable correlations to enhance imputation accuracy.

### 2.9 Statistical analysis

Descriptive statistics are presented as mean ± standard deviation or median (interquartile range) for continuous variables and as frequency (percentage) for categorical variables. To address selection bias in XNJI allocation, IPW was employed based on 25 baseline covariates (Table 3). Covariates were selected according to clinical relevance and prior literature (Lai, 2017; Xie, 2024), including demographics, baseline stroke severity, comorbidities, and acute treatment characteristics. Weights were truncated at the 99th percentile, and covariate balance was verified by standardized mean differences (SMD < 0.1).

**TABLE 3:** Baseline characteristics of AIS patients before and after IPW adjustment.

Variable		Before IPW			After IPW		
		Exposed group (n = 208)	Non-exposed group (n = 304)	SMD	Exposed group (n = 254)	Non-exposed group (n = 258)	SMD
Demographic data							
Age, years		66.0 [57.0, 74.0]	63.0 [55.0, 72.0]	0.197*	65.0 [56.0, 71.3]	64.0 [55.0, 73.0]	<0.001
Sex, n (%)	Female	84 (40.4)	85 (28.0)	0.264*	86.0 (33.9)	88.1 (34.1)	0.005
	Male	124 (59.6)	219 (72.0)		167.8 (66.1)	170.1 (65.9)	
BMI, kg/m^2^		25.7 [23.4, 28.0]	25.3 [23.4, 27.6]	0.089	25.6 [23.4, 27.6]	25.3 [23.4, 27.6]	0.01
History of underlying diseases							
Stroke, n (%)		66 (31.7)	99 (32.6)	0.018	84.7 (33.4)	85.8 (33.2)	0.003
CVD, n (%)		81 (38.9)	63 (20.7)	0.406*	73.3 (28.9)	73.3 (28.4)	0.011
Hypertension, n (%)		149 (71.6)	197 (64.8)	0.147*	163.9 (64.6)	164.1 (63.5)	0.021
Diabetes, n (%)		68 (32.7)	97 (31.9)	0.017	77.9 (30.7)	77.9 (30.2)	0.011
Hyperlipidemia, n (%)		31 (14.9)	43 (14.1)	0.022	36.0 (14.2)	37.8 (14.6)	0.013
Lifestyle risk factors							
Smoking, n (%)		92 (44.2)	173 (56.9)	0.256*	131.8 (51.9)	132.3 (51.2)	0.014
Alcohol drinking, n (%)		68 (32.7)	125 (41.1)	0.175*	95.3 (37.5)	95.3 (36.9)	0.013
Disease onset characteristics							
NIHSS score		4.0 [2.0, 9.0]	4.0 [2.0, 6.0]	0.209*	4.0 [2.0, 8.0]	4.0 [2.0, 7.0]	0.01
mRS score, n (%)		–	–	0.228*	–	–	0.069
0		7 (3.4)	22 (7.2)	​	12.0 (4.7)	15.6 (6.1)	​
1		94 (45.2)	139 (45.7)	​	113.1 (44.6)	114.0 (44.1)	​
2		43 (20.7)	68 (22.4)	​	56.7 (22.4)	56.0 (21.7)	​
3		39 (18.8)	43 (14.1)	​	42.5 (16.7)	40.6 (15.7)	​
4		15 (7.2)	23 (7.6)	​	20.7 (8.2)	23.1 (8.9)	​
5		10 (4.8)	9 (3.0)	​	8.7 (3.4)	8.9 (3.5)	​
TOAST_LAA, n (%)		Ref	Ref	Ref	Ref	Ref	Ref
TOAST_CE, n (%)		20 (9.6)	22 (7.2)	0.086	20.1 (7.9)	20.1 (7.8)	0.005
TOAST_SVO, n (%)		81 (38.9)	57 (18.8)	0.457*	79.5 (31.3)	79.5 (30.8)	0.012
TOAST_ODE, n (%)		2 (1.0)	1 (0.3)	0.079	1.0 (0.4)	1.0 (0.4)	0.001
TOAST_UDE, n (%)		6 (2.9)	3 (1.0)	0.138*	3.6 (1.4)	3.6 (1.4)	0.002
SBP, mmHg		159.0 [144.0, 171.5]	153.0 [136.0, 170.0]	0.215*	157.00 [142.0, 170.0]	154.0 [140.0, 170.0]	0.017
Abnormal laboratory indicators, n (%)							
ALT (vs. Reference range)		11 (5.3)	22 (7.2)	0.08	12.1 (4.8)	12.1 (4.7)	0.004
Cr (vs. Reference range)		38 (18.3)	51 (16.8)	0.039	45.1 (17.8)	45.6 (17.7)	0.003
NE% (vs. Reference range)		57 (27.4)	105 (34.5)	0.155*	79.9 (31.5)	80.7 (31.3)	0.005
D-dimer (vs. Reference range)		85 (40.9)	99 (32.6)	0.173*	93.2 (36.7)	93.2 (36.1)	0.013
Treatment during hospitalization							
Reperfusion therapy, n (%)		41 (19.7)	84 (27.6)	0.187*	61.2 (24.1)	62.5 (24.2)	0.002
Antithrombotic therapy, n (%)		177 (85.1)	283 (93.1)	0.259*	224.9 (88.6)	229.3 (88.8)	0.006
Antihypertensive therapy, n (%)		111 (53.4)	135 (44.4)	0.18*	122.5 (48.3)	122.7 (47.5)	0.015
Glucose lowering therapy, n (%)		62 (29.8)	107 (35.2)	0.115*	79.8 (31.5)	81.6 (31.6)	0.003
Lipid lowering therapy, n (%)		183 (88.0)	292 (96.1)	0.301*	235.2 (92.7)	239.7 (92.8)	0.005
Proprietary injection, n (%)		150 (72.1)	144 (47.4)	0.521*	147.9 (58.3)	150.5 (58.3)	<0.001
Herbal decoction, n (%)		156 (75.0)	92 (30.3)	1.002*	127.9 (50.4)	127.9 (49.5)	0.017

BMI, body mass index; CE, cardioembolism; CVD, cardiovascular disease; LAA, large-artery atherosclerosis; ODE, other determined etiology; Ref, reference category; SVO, small-vessel occlusion; TOAST, Trial of Org 10,172 in Acute Stroke Treatment; UDE, undetermined etiology; SMD, standardized mean difference.

* SMD > 0.1 indicates significant between-group imbalance at baseline.

For outcome analyses, weighted logistic regression (WLR) was used for END incidence and binary discharge outcomes (in-hospital mortality, inflammatory/coagulation abnormalities, and hepatic/renal dysfunction). Weighted generalized estimating equations (WGEE) were applied for longitudinal outcomes, including neurological recovery, SBP trajectories, and 3-month functional outcomes (mRS, BI). All outcome models were weighted by IPW based on 25 baseline covariates and further adjusted for in-hospital and post-discharge treatment factors. Unadjusted crude models were performed for comparative purposes. For longitudinal outcomes analyzed by weighted generalized estimating equations (WGEE), a non-significant overall group main effect accompanied by a significant time-by-group interaction solely indicates differential longitudinal recovery trajectories between the two groups, instead of a persistent overall intergroup difference in treatment effect. E-value was calculated to assess the robustness of the primary outcome against unmeasured confounding; a higher E-value represents lower vulnerability to potential residual confounding. All statistical analyses were performed using R version 4.3.1, and a two-sided P < 0.05 was defined as statistically significant.

### 2.10 Ethical considerations and reporting statement

The study protocol received ethical approval from the Ethics Committee of Dongzhimen Hospital, Beijing University of Chinese Medicine (approval No. DZMEC-KY-2019-153). Written informed consent was acquired from all subjects or their legal proxies prior to enrollment. This observational real-world cohort study was conducted in accordance with the STROBE reporting guidelines (Cuschieri, 2019).

## 3 Results

### 3.1 Patient enrollment and baseline characteristics

A total of 512 patients with AIS were enrolled, consisting of 208 (40.63%) receiving early XNJI (exposed group) and 304 (59.38%) managed with standard care alone (non-exposed group). Obvious baseline imbalances existed between the two groups before IPW weighting. Patients in the XNJI group were older (median age: 66.0 [57.0–74.0] vs. 63.0 [55.0–72.0] years, SMD = 0.197), had a higher proportion of cardiovascular comorbidities (38.9% vs. 20.7%, SMD = 0.406) and higher baseline NIHSS scores (median:4.0 [2.0–9.00] vs. 4.0 [2.0–6.00], SMD = 0.209), which reflected typical indication bias in real-world clinical practice (patients with more severe conditions were more likely to receive XNJI treatment). IPW incorporating 25 baseline confounders eliminated between-group imbalance, with all SMD <0.1 as specified in Table 3. All subsequent efficacy and safety analyses relied on the weighted cohort (n = 254 for the exposed group, n = 258 for the non-exposed group) to achieve comparable baseline profiles.

### 3.2 Outcome measures

#### 3.2.1 Efficacy indicators

##### 3.2.1.1 Primary efficacy indicator (END incidence)

The IPW-adjusted analysis (Table 4) demonstrated that XNJI administration was significantly correlated with a lower risk of END compared with standard care alone (adjusted odds ratio [aOR] = 0.46, 95% CI: 0.25–0.88; P = 0.018). This protective effect was more prominent than that observed in the unadjusted crude analysis (crude odds ratio [cOR] = 0.74), which was attributable to baseline selection bias. To evaluate robustness against potential unmeasured confounding, we calculated the E-value: an unmeasured confounder would need to increase both the probability of XNJI administration and END risk by 3.73–fold to fully explain away this association, further verifying the stability of the primary outcome.

**TABLE 4:** Association between XNJI exposure and END risk: IPW-adjusted WLR analysis.

Comparison groups	Weighted END incidence rate, %	cOR (95% CI)	aOR (95% CI)	E-value	*P*-value
Exposed vs. Non-exposed groups	10.15 vs. 19.58	0.74 (0.44–1.23)	0.46 (0.25–0.88)	3.73	0.018*

aOR, adjusted odds ratio (adjusted for 25 baseline covariates via IPW); cOR, crude odds ratio (unadjusted); E-value, sensitivity index for unmeasured confounding.

* P < 0.05.

##### 3.2.1.2 Secondary efficacy indicators

###### 3.2.1.2.1 Neurological recovery

**FIGURE 2:**
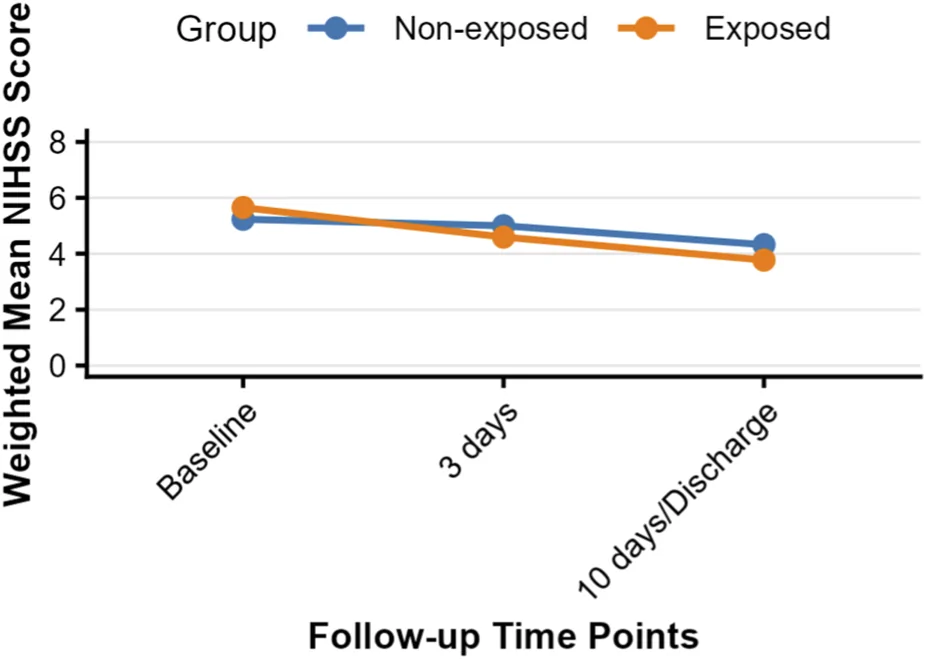
Longitudinal trajectories of NIHSS scores in AIS patients: IPW-adjusted WGEE analysis.

The IPW-adjusted WGEE model (Table 5) demonstrated a significant main effect of time (P < 0.001), indicating that neurological function gradually improved in both groups throughout the 10-day hospitalization period. Although the adjusted change in NIHSS score from baseline to Day 3 was not statistically significant (adjusted β = −0.23; 95% CI: −0.65 to 0.18; P = 0.266), NIHSS scores decreased significantly from Day 3 to Day 10 (adjusted β = −0.91; 95% CI: −1.47 to −0.35; P < 0.01). No significant overall main effect of group was observed (adjusted β = 0.17; 95% CI: −0.98 to 1.33; P = 0.769), suggesting there was no persistent overall difference in neurological function between the two groups. Significant time-by-group interactions were detected at both Day 3 and Day 10 (Day 3: adjusted β = −0.82; 95% CI: −1.45 to −0.19; P = 0.011; Day 10: adjusted β = −0.97; 95% CI: −1.76 to −0.18; P = 0.016), indicating divergent neurological recovery trajectories between groups: patients receiving XNJI had a faster recovery of neurological function during hospitalization. These trends were consistent with those presented in Figure 2. Crude (unadjusted) effect estimates are also listed in Table 5 for comparison.

**TABLE 5:** Longitudinal changes in NIHSS scores during hospitalization: IPW-adjusted WGEE analysis.

Effect term	Crude β (95% CI)	Adjusted β (95% CI)	*P*-value
Intercept	4.96 (4.43–5.48)	4.56 (3.67–5.45)	<0.001***
Time effect (Day 3 vs. Baseline)	−0.30 (−0.61 to −0.00)	−0.23 (−0.65 to 0.18)	0.266
Time effect (Day 10 vs. Day 3)	−1.04 (−1.44 to −0.64)	−0.91 (−1.47 to −0.35)	<0.01**
Group effect (exposed vs. Non-exposed)	1.14 (0.21–2.07)	0.17 (−0.98–1.33)	0.769
Time × exposure interaction (Day 3)	−1.08 (−1.72 to −0.44)	−0.82 (−1.45 to −0.19)	0.011*
Time × exposure interaction (Day 10)	−1.10 (−1.84 to −0.36)	−0.97 (−1.76 to −0.18)	0.016*

Crude β, unadjusted effect estimate; Adjusted β, effect estimate adjusted for 26 covariates (25 IPW-adjusted baseline covariates and in-hospital rehabilitation therapy).

* P < 0.05.

** P < 0.01.

*** P < 0.001.

###### 3.2.1.2.2 Functional prognosis functional prognosis

####### 3.2.1.2.2.1 Functional Independence (mRS ≤ 2)

**FIGURE 3:**
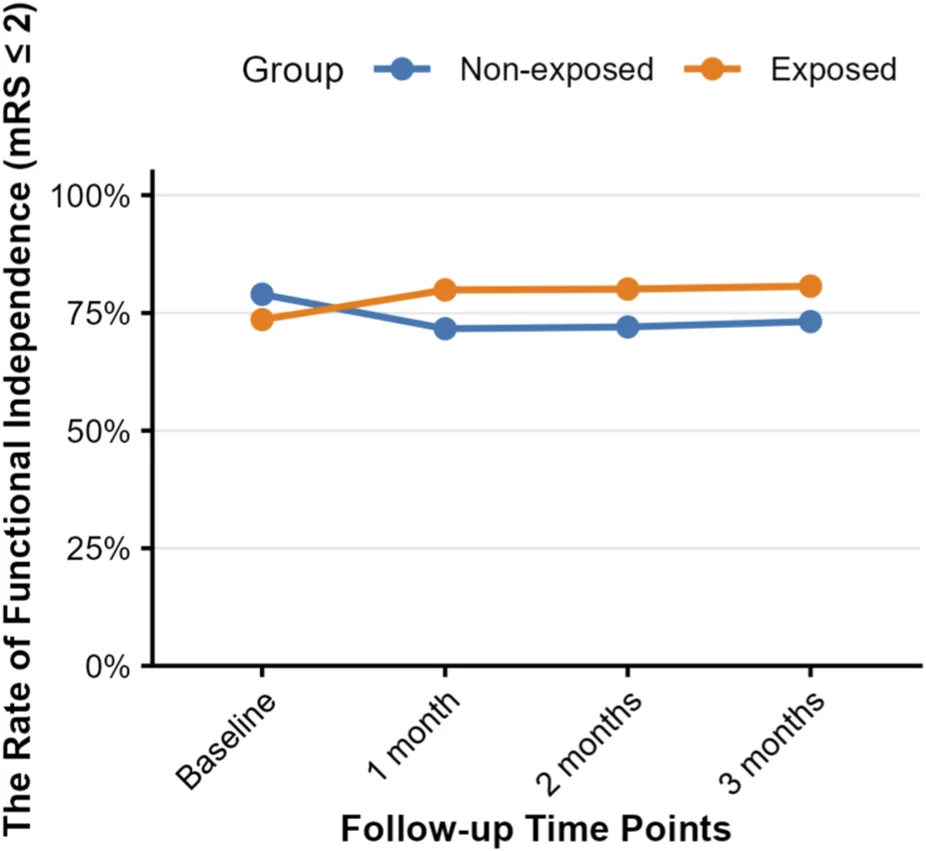
Longitudinal changes in functional independence (mRS ≤ 2) among AIS patients: IPW-adjusted WGEE analysis.

The IPW-adjusted WGEE model (Table 6) was used to analyze longitudinal changes in functional independence (mRS ≤ 2). For the overall cohort, the rate of functional independence decreased significantly from baseline to the 1-month follow-up (aOR = 0.64, 95% CI: 0.44–0.94, P = 0.023) and from the 1-month follow-up to the 2-month follow-up (aOR = 0.66, 95% CI: 0.44–0.98, P = 0.039), and the declining trend gradually plateaued at the 3-month follow-up (aOR = 0.70, 95% CI: 0.47–1.04, P = 0.079). There was no significant overall group main effect throughout the 3-month follow-up (aOR = 0.76, 95% CI: 0.43–1.37, P = 0.367). Nevertheless, significant time-by-group interactions were observed at the 1-, 2-, and 3-month follow-ups (all P < 0.05), indicating that the two groups exhibited divergent functional independence trajectories over the 3-month follow-up (Figure 3). After multivariate adjustment, the exposed group had significantly higher odds of achieving functional independence at all three follow-up time points (1 month: aOR = 2.25; 2 months: aOR = 2.23; 3 months: aOR = 2.18). In contrast, crude unadjusted analyses (Supplementary Table S1) found no intergroup differences at any time point (all P > 0.05). This discrepancy was mainly attributed to baseline imbalance: the exposed group had a lower baseline rate of functional independence and more severe neurological deficits, which obscured the actual between-group difference in unadjusted analyses. Notably, the significant interaction effects only demonstrate differences in long-term recovery trajectories, rather than a sustained overall superiority of XNJI over standard care.

**TABLE 6:** Effect of XNJI on functional independence (mRS ≤ 2) over 3-month follow-up: IPW-adjusted WGEE analysis.

Effect term	aOR (95% CI)	*P*-value
Intercept	8.18 (3.13–21.40)	<0.001***
Time effect (1 month vs. baseline)	0.64 (0.44–0.94)	0.023*
Time effect (2 vs. 1 month)	0.66 (0.44–0.98)	0.039*
Time effect (3 vs. 2 months)	0.70 (0.47–1.04)	0.079
Group effect (exposed vs. Non-exposed)	0.76 (0.43–1.37)	0.367
Time × exposure interaction (1 month)	2.25 (1.12–4.56)	0.024*
Time × exposure interaction (2 months)	2.23 (1.09–4.59)	0.028*
Time × exposure interaction (3 months)	2.18 (1.06–4.47)	0.033*

aOR, adjusted odds ratio via WGEE (adjusted for 25 IPW-weighted baseline covariates and seven treatment-related covariates).

* P < 0.05.

*** P < 0.001.

####### 3.2.1.2.2.2 Independent activities of daily living (ADL) ability (BI ≥ 90)

**FIGURE 4:**
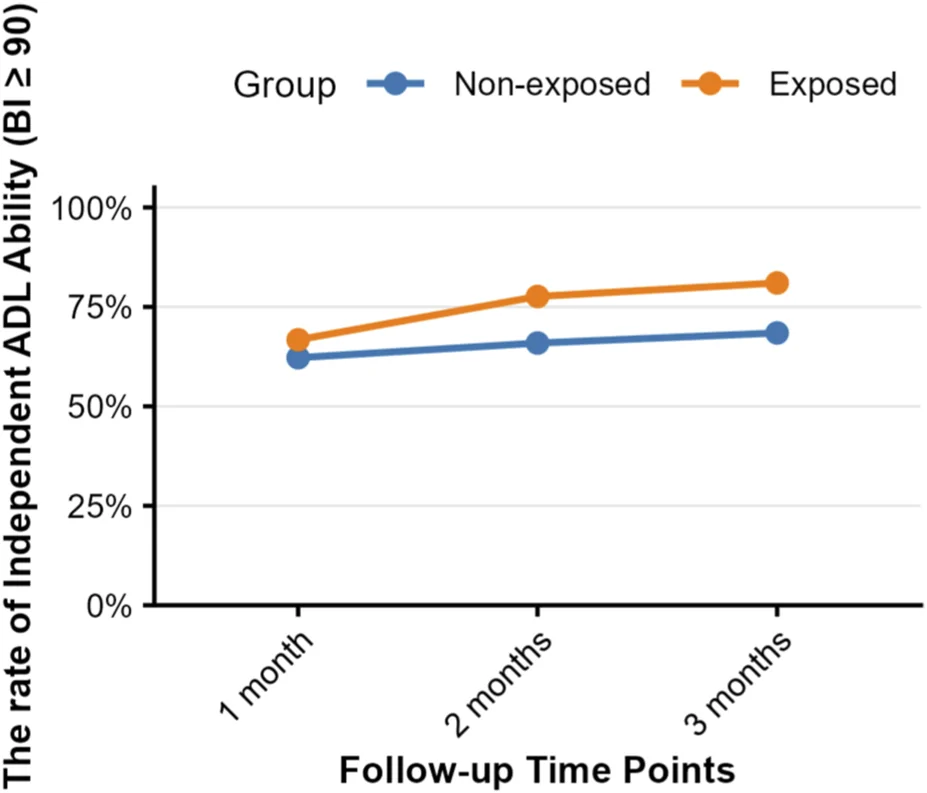
Longitudinal changes in independent activities of daily living (BI ≥ 90) among AIS patients: IPW-adjusted WGEE analysis.

The IPW-adjusted WGEE model (Table 7) revealed a significant main effect of time: the odds of independent ADL increased from the 1-month follow-up to the 2-month follow-up (aOR = 1.20, 95% CI: 0.98–1.48, P = 0.079) and continued to rise from 2 months to 3 months (aOR = 1.37, 95% CI: 1.05–1.80, P = 0.021). The overall group main effect was not significant (exposed vs. non-exposed: aOR = 1.45, 95% CI: 0.84–2.49, P = 0.182). However, significant time × group interactions were detected: the exposed group had significantly higher odds of independent ADL at 2 months (aOR = 1.49, 95% CI: 1.02–2.17, P = 0.040) and 3 months after stroke onset (aOR = 1.62, 95% CI: 1.03–2.53, P = 0.035) (Figure 4). Unadjusted crude analyses (Supplementary Table S2) showed no intergroup differences in ADL ability across all follow-up time points (all P > 0.05), which was inconsistent with the results after IPW adjustment. Again, these significant interaction effects solely indicate that the two groups presented different recovery trajectories of daily living ability over time, with no evidence of an overall group difference.

**TABLE 7:** Effect of XNJI on independent ADL (BI ≥ 90) over 3-month follow-up: IPW-adjusted WGEE analysis.

Effect term	aOR (95% CI)	*P*-value
Intercept	4.44 (1.72–11.45)	<0.01**
Time effect (2 vs. 1 month)	1.20 (0.98–1.48)	0.079
Time effect (3 vs. 2 months)	1.37 (1.05–1.80)	0.021*
Group effect (exposed vs. Non-exposed)	1.45 (0.84–2.49)	0.182
Time × exposure interaction (2 months)	1.49 (1.02–2.17)	0.040*
Time × exposure interaction (3 months)	1.62 (1.03–2.53)	0.035*

aOR, adjusted odds ratio derived from WGEE (adjusted for 25 IPW-weighted baseline covariates and seven treatment-related covariates).

* P < 0.05.

*** P < 0.001.

###### 3.2.1.2.3 Inflammation and coagulation regulation

The IPW-adjusted WLR model (Table 8) demonstrated that, compared to the non-exposed group, the exposed group exhibited a lower risk of neutrophil percentage (NE%) abnormalities (cOR = 0.49, 95% CI: 0.26–0.90; aOR = 0.52, 95% CI: 0.25–1.05, P = 0.02) and D-dimer abnormalities (cOR = 0.56, 95% CI: 0.34–0.91; aOR = 0.53, 95% CI: 0.31–0.91, P = 0.02).

**TABLE 8:** Abnormalities in inflammatory and coagulation markers: IPW-adjusted WLR analysis.

Variable	Exposed vs. Non-exposed group	cOR (95% CI)	aOR (95% CI)	*P*-value
NE%	14.70% vs. 26.20%	0.49 (0.26–0.90)	0.52 (0.25–1.05)	0.02*
D-dimer	24.90% vs. 37.40%	0.56 (0.34–0.91)	0.53 (0.31–0.91)	0.02*

cOR, crude odds ratio (unadjusted); aOR, adjusted odds ratio via WLR (adjusted for 25 IPW-weighted baseline covariates and in-hospital rehabilitation therapy).

* P < 0.05.

#### 3.2.2 Safety indicators

##### 3.2.2.1 In-hospital mortality and cause of death composition

The IPW-adjusted WLR model (Table 9) showed that the weighted in-hospital mortality rate was 0.5% in the exposed group and 2.00% in the non-exposed group. Although the in-hospital mortality rate was numerically lower in the exposed group, the difference was not statistically significant (aOR = 0.27, 95% CI: 0.05–1.58, P = 0.148). The statistical power for mortality analysis was limited due to the small number of death events. The unadjusted crude analysis yielded similar non-significant results (cOR = 0.48, 95% CI: 0.07–2.12). The causes of death differed between the two groups. There were 2 deaths in the exposed group, including one case of pulmonary infection and one case of cerebral hernia. A total of 6 deaths occurred in the non-exposed group, consisting of 3 cases of cardiogenic shock, 2 cases of pulmonary infection, and 1 case of cerebral hernia complicated with hemorrhagic transformation (Table 9).

**TABLE 9:** In-hospital mortality and causes of death: IPW-adjusted WLR analysis.

Group	Pulmonary infection	Cardiogenic shock	Cerebral hernia formation	Weighted in-hospital mortality rate	cOR (95% CI)	aOR (95% CI)	*P*-value
Exposed vs. Non-exposed group	1 vs. 2	0 vs. 3	1 vs. 1	0.50% vs. 2.00%	0.48 (0.07–2.12)	0.27 (0.05–1.58)	0.148

cOR, crude odds ratio (unadjusted); aOR, adjusted odds ratio via WLR (adjusted for 25 IPW-weighted baseline covariates and in-hospital rehabilitation therapy).

##### 3.2.2.2 SBP trajectory

**FIGURE 5:**
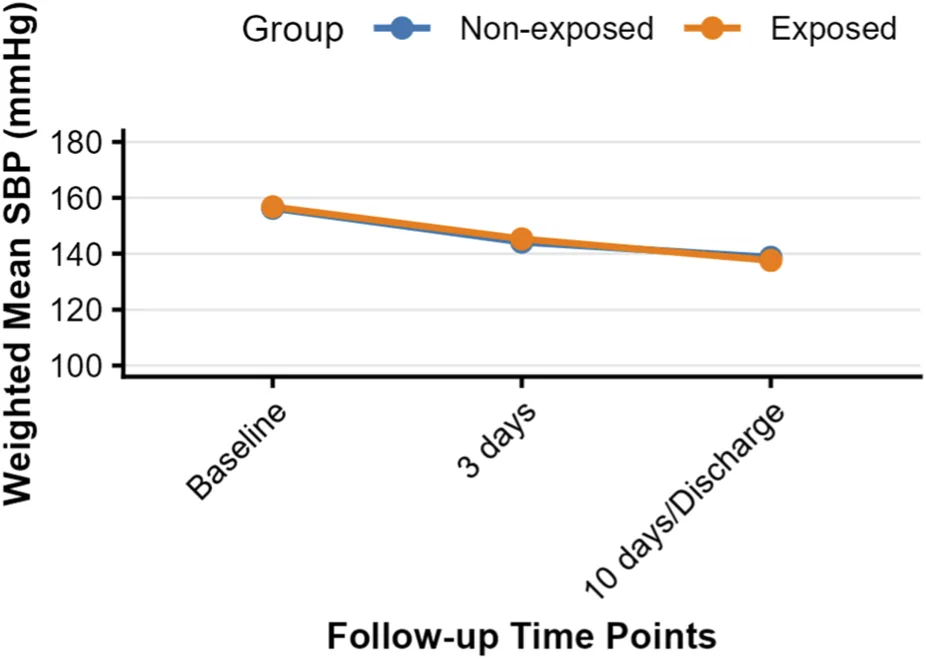
Systolic blood pressure (SBP) trajectories during hospitalization in AIS patients: IPW-adjusted WGEE analysis.

The IPW-adjusted WGEE model (Table 10) revealed a significant downward trend in SBP for both patient groups during hospitalization. Compared to baseline, SBP decreased by −12.12 mmHg (95% CI: −15.91 to −8.33; P < 0.001) at Day 3, with a further reduction of −17.59 mmHg (95% CI: −21.10 to −14.07; P < 0.001) observed at Day 10 compared to Day 3, culminating in a total adjusted reduction of 29.00 mmHg. Critically, no statistically significant difference in SBP levels was observed between the exposed and non-exposed groups (main group effect: β = 0.66 mmHg, 95% CI: −5.60 to 6.92; P = 0.836). Furthermore, no significant group-time interaction effects were detected in the SBP trajectory (Day 3 interaction term: β = 0.64, 95% CI: −5.26 to 6.54; P = 0.831; Day 10 interaction term: β = −1.58, 95% CI: −6.99 to 3.82; P = 0.565). Figure 5 illustrates the highly parallel dynamic SBP trends in both groups.

**TABLE 10:** SBP trajectories during hospitalization: IPW-adjusted WGEE analysis.

Effect term	Crude β (95% CI)	Adjusted β (95% CI)	*P*-value
Intercept	155.35 (152.25–158.46)	156.75 (152.59–160.91)	<0.001***
Time effect (Day 3 vs. baseline)	−9.34 (−12.02 to −6.66)	−12.12 (−15.91 to −8.33)	<0.001***
Time effect (Day 10 vs. Day 3)	−14.79 (−17.37 to −12.21)	−17.59 (−21.10 to −14.07)	<0.001***
Group effect (exposed vs. Non-exposed)	4.66 (−0.05–9.38)	0.66 (−5.60–6.92)	0.836
Time × exposure interaction (Day 3)	−5.94 (−10.54 to −1.35)	0.64 (−5.26–6.54)	0.831
Time × exposure interaction (Day 10)	−6.80 (−10.89 to −2.71)	−1.58 (−6.99 to 3.82)	0.565

Crude β: Unadjusted effect estimate; Adjusted β: Effect estimate derived from WGEE (adjusted for 25 IPW-weighted baseline covariates and in-hospital rehabilitation therapy).

*** P < 0.001.

##### 3.2.2.3 Hepatic/renal function abnormalities

The IPW-adjusted WLR model (Table 11) identified no statistically significant between-group differences in the incidence of renal and hepatic dysfunction during hospitalization (Creatinine [Cr]: aOR = 1.04, 95% CI: 0.49–2.18, P = 0.915; Alanine aminotransferase [ALT]: aOR = 1.09, 95% CI: 0.53–2.21, P = 0.646). Unadjusted analyses yielded consistent non-significant findings. These results confirm that short-term administration of XNJI does not increase the risk of hepatic or renal dysfunction in patients with AIS.

**TABLE 11:** Abnormalities in hepatic and renal safety indicators: IPW-adjusted WLR analysis.

Variable	Exposed vs. Non-exposed group	cOR (95%CI)	aOR (95%CI)	*P*-value
Cr abnormality (>ULN)	17.00% vs. 16.50%	0.90 (0.51–1.56)	1.04 (0.49–2.18)	0.915
ALT abnormality (>ULN)	16.10% vs. 14.10%	1.39 (0.78–2.48)	1.09 (0.53–2.21)	0.646

cOR, crude odds ratio (unadjusted); aOR, adjusted odds ratio via WLR (adjusted for 25 IPW-weighted baseline covariates and in-hospital rehabilitation therapy); ULN, upper limit of normal.

## 4 Discussion

This multicenter prospective observational cohort investigated XNJI administration within 24 h of AIS onset. All findings represent observational associations rather than causal relationships. This study adds real-world evidence for early XNJI application, offering clinical references for AIS patients who are unable to receive reperfusion therapy due to time limits and bleeding risks. While prior studies mostly adopted a 72-h intervention window, our 24-h window aligns better with prehospital emergency practice. Standardized study definitions and IPW were used to balance baseline characteristics and reduce selection bias, strengthening the credibility of association analyses.

END is a critical acute complication after ischemic stroke and strongly predicts long-term disability and mortality, representing a core primary endpoint for evaluating acute adjuvant therapeutic strategies. In this study, early XNJI administration within 24 h of AIS onset was observed to be associated with a lower risk of END. Notably, an obvious discrepancy was observed between unadjusted cOR (cOR = 0.74) and the IPW-aOR (aOR = 0.46) regarding END risk. This difference was attributed to typical indication bias in real-world clinical practice. Patients with higher baseline NIHSS scores and greater cardiovascular comorbidity burden were more likely to receive XNJI adjuvant treatment, resulting in baseline imbalance and underestimating the genuine protective association in unadjusted analyses. After IPW achieved excellent covariate balance with all SMD<0.1, E-value analysis further confirmed moderate-to-high robustness against unmeasured confounding, indicating that any unobserved confounder would need to exert nearly fourfold influence on both XNJI exposure and END risk to reverse the present findings, which is clinically implausible in routine AIS management.

Longitudinal trajectory analysis indicated no significant overall main effect of XNJI administration on neurological and functional recovery; instead, significant time-treatment interactions were observed. Early XNJI administration was correlated with accelerated neurological recovery at Days 3 and 10 post-onset, higher odds of functional independence (mRS ≤ 2) at the 1-, 2-, and 3-month follow-ups, and an increased likelihood of independent ADL (BI ≥ 90) at the 2- and 3-month follow-ups. These observational associations are in accordance with findings from previous meta-analyses suggesting that XNJI can alleviate neurological deficits and improve functional outcomes (Chen and Gu, 2017; Wang et al., 2022). The lower rates of NE% and D-dimer abnormalities observed in the exposed group were also correlative findings, aligning with previous pharmacological studies and meta-analyses that reported anti-inflammatory and anticoagulant properties of XNJI (Peng et al., 2014; Liu et al., 2024). Previous pharmacological studies have shown that the active ingredients of XNJI can regulate inflammation and coagulation function (Liu, 2022; Wu and Xu, 2020). Dynamic changes of inflammatory and coagulation indicators during hospitalization were partially correlated with the trend of neurological function recovery and END occurrence. It is speculated that the regulatory effect of XNJI on inflammation and coagulation may be one of the potential mechanisms for reducing the risk of early neurological deterioration and promoting functional recovery. However, such ecological consistency does not imply direct mechanistic confirmation in this observational setting. Further targeted basic and clinical researches are needed to verify the causal chain.

In terms of safety outcomes, in-hospital mortality was numerically lower in the exposed group with no statistical significance, which may be attributed to the small number of death events and insufficient statistical power. No drug-related fatal adverse events occurred in the XNJI group. There were no significant between-group differences in the dynamic changes of SBP during hospitalization. Meanwhile, the incidence of hepatorenal dysfunction was similar in both groups, indicating that short-term early administration of XNJI did not increase the risk of hemodynamic instability or hepatorenal damage. These findings are consistent with previous post-marketing safety evaluations and meta-analyses (Zhang B. H. et al., 2021; Zhao et al., 2025).

This study has notable methodological strengths, including prospective multicenter enrollment, standardized outcome assessment, rigorous study design, IPW-based baseline covariate balancing (all SMD < 0.1), E-value assessment for confounding robustness, and a 3-month long-term follow-up. These strengths ensure reliable real-world evidence and good clinical reference value. Nevertheless, several limitations should be acknowledged. First, we balanced baseline features via single-time static IPW and additionally adjusted for in-hospital and post-discharge concomitant treatment covariates; however, complex time-varying confounding could not be fully ruled out. We lacked real-time dynamic data (daily rehabilitation intensity, post-discharge medication adherence, individualized clinical adjustments), which may induce residual confounding bias for the observed associations. Second, XNJI administration was individualized in routine clinical practice, and we did not conduct dose-response subgroup analyses. Third, all participants were recruited from tertiary hospitals in Beijing, which limits the generalizability of our findings to other medical institutions and regional populations. Fourth, the number of in-hospital deaths was small, resulting in insufficient statistical power for mortality analysis; moreover, we did not systematically collect detailed adverse events of XNJI, leading to an incomplete safety evaluation. Fifth, as an observational cohort study, this work only reveals associations rather than causal relationships. Despite multiple measures to reduce selection bias, residual confounding still exists, so all conclusions should be interpreted cautiously and the evidence level is inferior to that of randomized controlled trials.

In summary, this multicenter prospective real-world cohort study showed that early XNJI administration within 24 h of AIS onset was associated with lower END risk. Patients receiving early XNJI intervention recovered faster in both acute neurological function and long-term functional prognosis, yet no consistent overall therapeutic benefit spanning all follow-up time points was detected relative to standard stroke care alone. We detected no meaningful associations between XNJI use and elevated risks of in-hospital death, abnormal SBP trends, or hepatorenal dysfunction. For a substantial number of AIS patients who cannot receive reperfusion therapy, these real-world findings provide preliminary references for early XNJI use. Large-scale nationwide multicenter RCTs are therefore warranted to validate the observed associations, explore optimal dosage regimens, and formulate unified clinical application standards for XNJI in emergency management of acute stroke.

## Data Availability

The raw data supporting the conclusions of this article will be made available by the authors, without undue reservation.
