# Supplementary material for: Effectiveness and safety of xingnaojing injection within 24 hours for acute ischemic stroke: a multicenter prospective cohort study (TRACE)
Source: Front Pharmacol. 2026 Aug 12;17:1697607. doi: 10.3389/fphar.2026.1697607 (PMC13506927; doi:10.3389/fphar.2026.1697607)
Supplement: Supplementary file 2 [file Table1.docx]

Supplementary Material

# 1 Supplementary Tables

**Supplementary Table 1. Crude Odds Ratio (cOR) Analysis of Functional Independence (mRS≤2) : Unadjusted Comparisons**

| Follow-up time | Exposed group | Non-exposed group | cOR (95% CI) | *P*-value |
| --- | --- | --- | --- | --- |
| Baseline | 135/187 (72.2%) | 196/246 (79.7%) | 0.66 (0.42–1.03) | 0.070 |
| 1 month after onset | 145/187 (77.5%) | 184/246 (74.8%) | 1.16 (0.75–1.83) | 0.508 |
| 2 months after onset | 146/187 (78.1%) | 186/246 (75.6%) | 1.15 (0.73–1.81) | 0.548 |
| 3 months after onset | 147/187 (78.6%) | 185/246 (75.2%) | 1.21 (0.77–1.92) | 0.407 |

cOR, crude odds ratio(unadjusted).

**Supplementary Table 2. Crude Odds Ratio (cOR) Analysis of Independent Activities of Daily Living (BI≥90) : Unadjusted Comparisons**

| Follow-up time | Exposed group | Non-exposed group | cOR (95% CI) | *P*-value |
| --- | --- | --- | --- | --- |
| 1 month after onset | 118/187 (63.1%) | 163/246 (66.3%) | 0.87 (0.59–1.30) | 0.495 |
| 2 months after onset | 138/187 (73.8%) | 173/246 (70.3%) | 1.19 (0.78–1.83) | 0.427 |
| 3 months after onset | 147/187 (78.6%) | 177/246 (72.0%) | 1.43 (0.92–2.25) | 0.115 |

cOR, crude odds ratio(unadjusted).
